# Bioactive (Co)oligoesters as Potential Delivery Systems of p-Anisic Acid for Cosmetic Purposes

**DOI:** 10.3390/ma13184153

**Published:** 2020-09-18

**Authors:** Magdalena Martinka Maksymiak, Magdalena Zięba, Arkadiusz Orchel, Monika Musiał-Kulik, Marek Kowalczuk, Grazyna Adamus

**Affiliations:** 1 Centre of Polymer and Carbon Materials, Polish Academy of Sciences, M. Curie-Skłodowskiej 34 Str., 41–819 Zabrze, Poland; mzieba@cmpw-pan.edu.pl (M.Z.); mmusial@cmpw-pan.edu.pl (M.M.-K.); marek.kowalczuk@cmpw-pan.edu.pl (M.K.); 2 Department of Biopharmacy, Faculty of Pharmaceutical Sciences in Sosnowiec, Medical University of Silesia, Katowice, Poland, Jednosci 8 Str., 41–208 Sosnowiec, Poland; aorchel@sum.edu.pl

**Keywords:** polyhydroxyalkanoates, oligo(3-hydroxy-3-(4-methoxybenzoyloxymethyl)propionate), bioactive (co)oligoesters, p-anisic acid derivatives, hydrolytic degradation, cosmetic delivery system, ESI-MS, multistage mass spectrometry

## Abstract

This article reports the studies on bioactive (co)oligoesters towards their use as controlled delivery systems of p-anisic acid. The objects of the study were oligo[3-hydroxy-3-(4-methoxybenzoyloxymethyl)propionate], (p-AA-CH_2_-HP)_n_ oligoester, and oligo[(3-hydroxy-3-(4-methoxybenzoyloxymethyl)propionate)-co-(3-hydroxybutyrate)] [(p-AA-CH_2_-HP)_x_-co-(HB)_y_ (co)oligoesters containing p-anisic acid moiety (p-AA, as the bioactive end and side groups) connected to the polymer backbone through the susceptible to hydrolysis ester bonds. A thorough insight into the hydrolysis process of the bioactive (co)oligoesters studied has allowed us to determine the release profile of p-AA as well as to identify polymer carrier degradation products. The p-AA release profiles determined on the basis of high-performance liquid chromatography (HPLC) measurements showed that the release of the bioactive compound from the developed (co)oligoester systems was regular and no burst effect occurred. Biological studies demonstrated that studied (homo)- and (co)oligoesters were well tolerated by HaCaT cells because none of them showed notable cytotoxicity. They promoted keratinocyte growth at moderate concentrations. Bioactive (co)oligoesters containing p-anisic acid moiety had somewhat decreased cell proliferation at the highest concentration (100 µg/mL). The important practical inference of the current study is that the (co)oligoesters developed have a relatively large load of the biologically active substance (p-AA) per polymer macromolecule, which unlocks their potential application in the cosmetic industry.

## 1. Introduction

Natural antioxidants offer a host of benefits, especially for our health and general well-being, including anti-aging, anti-carcinogenic, anti-inflammatory, and anti-microbial properties, which promote a growing use in cosmetic products. The antioxidant activities of natural antioxidants have been largely ascribed to the presence of phenolic content [1,2].

Additionally, antioxidants possess very strong preservative properties and they may prevent lipid oxidation in cosmetic products. Besides, sunlight, air and vehicle pollution, and other environmental factors produce free radicals, which could be neutralized by antioxidants. The use of antioxidants in cosmetics aims to create a barrier that helps protect the skin against free radicals produced by oxidative stressors [3].

Among phenols, which are a well-known group of biologically active compounds, the best known are flavonoids, tannins, and phenolic acids. The last ones belong to aromatic secondary plant metabolites, are widespread in vegetation, and provide a wealth of anti-aging benefits. They can be readily absorbed in the human body and demonstrate strong preserving properties, which include anti-bacterial and anti-fungal effects [4]. The main function of phenolic antioxidants is retardation oxidation of unsaturated oils that may affect the final color and smell of cosmetic products [5].

p-Anisic acid (4-Methoxybenzoic acid) reveals antioxidant, anti-inflammatory, and anti-tumor properties, and is a proven antiseptic, which makes it suitable as a preservative in cosmetic products [5]. Over the past few years, p-AA became progressively substantial when used as a multi-functional material in the cosmetics and food sectors [6]. Among various cosmetic ingredients with antimicrobial properties, there are some commercially available ones (p-anisic acid and levulinic acid), which were found in number in several herbs (e.g., *Pimpinella anisum*) and as a by-product in the preparation of diosgenin from wild yam (*Dioscorea villosa*) [7].

The other developed cosmetic products, consisting of these alternative anti-microbial substances, are characterized as preservative-free or naturally preserved cosmetics [8].

Nevertheless, antioxidants encounter some difficulties, especially concerning the stability problems when they penetrate the transdermal barrier [9]. The use of controlled release systems in cosmetics contributes to the improvement of antioxidants’ skin penetration. Moreover, it is a further challenge that the developed delivery systems (DSs) are readily integrated into the final cosmetic product formulation, thus leading to a consistent product for the customers’ use. Furthermore, the use of DS enhances the protection of sensitive active ingredients and guarantees their targeted controlled release. It was proved that cosmetic DSs demonstrate a positive impact on the permeation of the bioactive compounds throughout the skin layers, and thus the concentration of the active species may be well controlled in both the obtained formulation and in the skin, respectively. So far, several types of DSs (e.g., niosomes, transfersomes, liposomes, lipid nanoparticles, polymeric microparticles, and nanoparticles) have been applied in cosmetic products. Skin interaction with the various DSs depends mostly on such factors as size, flexibility, and composition [10].

Many works have demonstrated that the improvement of the skin penetration of indigenously applied drugs can be achieved using particle-based formulations. However, a more rational approach needs to be considered when the transfer of the promising results to the clinical application is planned. Moreover, the different properties of diseased skin and the fate of these polymeric materials should be taken into account. Ideally, depending on the nature of the polymer, decomposition of a biodegradable polymer into non-toxic products and its degradation in a controlled manner after topical applications on the skin surface is highly desired [11].

The emergence of a broad spectrum of biodegradable polymers dedicated specifically for biomedical applications has resulted from the development of macromolecules with well determined structure and properties. The synthetic aliphatic polyesters possess a unique position among biodegradable polymers. They show the possibility of many health-related applications thanks to their good biodegradability and biocompatibility [12,13].

β-Lactones are considered as attractive substrates in organic synthesis [14] that can be used as monomers for the synthesis of aliphatic polyesters via ring-opening polymerization (ROP) [15,16]. Quite recently, selective and highly active catalysts that might be used for epoxide carbonylation have been described [17,18,19]. Thus, a new source of bioactive β-lactone monomers that contain in their structure a biologically active substance has been established. They proved to be very valuable and helpful for the synthesis of new polyesters, including polymeric DS with bioactive moieties. Polymer-bioactive compound conjugates offer countless benefits over free drugs such as increased water solubility, controlled and targeted drug delivery, more bioavailability, and thus enhanced therapeutic efficacy [20].

In our previous studies, we provided an overview of the approach for synthesizing bioactive (co)oligoesters on the basis of anionic homo- and co-polymerization of selected β-substituted β-lactones that contain bioactive species selectively chosen from compounds commonly used in cosmetic industry. The molecular-level structure of the obtained bioactive (co)oligoesters, as well as their abilities in the prospective application in cosmetology, were also demonstrated [21,22,23,24,25].

In the present contribution, we present a further extension study on bioactive (co)oligoesters that contain p-anisic acid moieties bound along oligomer chains. Considering the future use of developed biomaterials as controlled DS of p-AA, the current research has been focused on detailed studies of hydrolytic degradation of the resulting bioactive (co)polyesters.

The results of studies on the hydrolytic degradation of the obtained materials allowed us to gain full knowledge of the hydrolysis process and examine the p-AA release profile. In addition, applying mass spectrometry has enabled to identify the products of degradation of the biomaterials studied and determine their molecular structure.

Moreover, both bioactive (homo)- and (co)oligoesters were found to be cytocompatible when in contact with human HaCaT cells and they seem to be optimal to support keratinocyte functions. Especially, (p-AA-CH_2_-HP)_n_ oligoester did not exert any inhibitory impact on cell growth, even at a relatively high concentration. Incubation with (co)oligoester had beneficial effects on HaCaT cells at concentrations below 100 µg/mL.

## 2. Materials and Methods

### 2.1. Materials

p-Anisic acid sodium salt (sodium p-anisate, 4-methoxybenzoic acid sodium salt for synthesis, Merck KGaA, Darmstadt, Germany) was used without further treatment. LC-MS grade methanol, chloroform, acetonitrile and 0.1% formic acid in water were purchased from Merck (Merck KGaA, Darmstadt, Germany). (*R,S*)-β-Butyrolactone (β-BL, Sigma-Aldrich Chemie GmbH, Steinheim, Germany) was distilled over CaH_2_ and the fraction boiling at 56 °C (9 mmHg) was collected. The 4-Methoxybenzoyloxymethythylpropiolactone (p-AA-CH_2_-PL) was synthesized at the Institute of Organic Chemistry, Polish Academy of Sciences by carbonylation of the respective epoxide under ambient CO pressure [26].

The oligo(3-hydroxy-3-(4-methoxybenzoyloxymethyl)propionate) (p-AA-CH_2_-HP)_n_ oligoesters (sample 1; M_n,GPC_ = 700, M_w_/M_n_ = 1.15) and oligo[(3-hydroxy-3-(4-methoxybenzoyloxymethyl)-propionate)-co-(3-hydroxybutyrate)], [(p-AA-CH_2_-HP)_x_-co-(HB)_y_] (co)oligoesters (sample 2; M_n,GPC_ = 900 M_w_/M_n_ = 1.23) were obtained via anionic ring-opening oligomerization of (p-AA-CH_2_-PL) and via anionic ring-opening (co)oligomerization of β-BL with p-AA-CH_2_-PL, respectively. The synthesis was performed according the procedure described in [25]. The resulting (co)oligoesters were purified and characterized by ^1^H nuclear magnetic resonance (^1^H NMR), gel permeation chromatography (GPC), and multistage mass spectrometry (ESI-MS^n^).

### 2.2. Measurements

#### 2.2.1. Performance of Electrospray Mass Spectrometry (ESI-MS^n^) Analyses

Electrospray mass spectra were acquired by directly infusing the polyester sample into the ESI source of a Finnigan LCQ ^TM^ ion trap mass spectrometer (Finnigan, San Jose, CA, USA) with a syringe pump at a flow rate of 10 mL min^−1^. Nitrogen was used as the nebulising gas. The LCQ ESI source was operated at 4.5 kV, and the capillary heater was set to 200 °C. The polyester samples were diluted in a chloroform/methanol system (1:1; *v/v*). For fragmentation experiments, the ions of interest were isolated monoisotopically in the ion trap and were collisionally activated. The helium damping gas that was present in the mass analyzer acted as the collision gas. The ring electrode radio-frequency (RF) amplitude with a significant voltage range was set to a value that caused the peak height of the molecular ion to decrease by at least 50%. The analyses were accomplished in the negative-ion mode.

#### 2.2.2. Performing of HPLC Analyses

**Figure 1:**
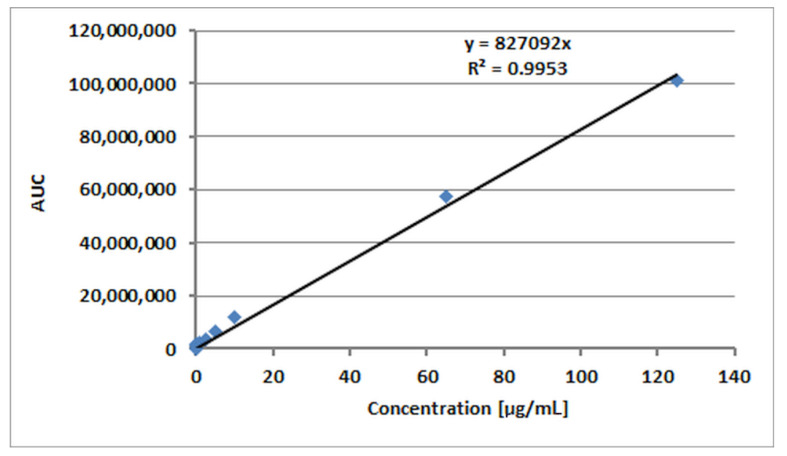
High-performance liquid chromatography (HPLC) calibration curve for p-anisic acid.

Qualitative and quantitative analyses by high-performance liquid chromatography (HPLC—VWR/Hitachi LaChrom Elite^®^, Tokyo, Japan) on the release of p-AA from the respective (homo)- and (co)oligoesters were performed. The samples were filtered through syringe filters Iso-Disc™ (0.2 μm, Supelco^®^) (Sigma-Aldrich Chemie GmbH, Steinheim, Germany) and separated on a LiChrospher^®^ RP-18 column (250 mm × 4 mm, 5 μm, Merck) protected by guard column LiChrospher^®^ RP-18 (4 mm × 4 mm, 5 μm, Merck KGaA, Darmstadt, Germany). The mobile phase consisted of 0.1% formic acid in water and acetonitrile. Table 1 shows the solvent gradient used for separation. The flow rate was set at 1 mL/min. The evaluation was performed using diode array detector (DAD) at 254 nm. The samples were analyzed in triplicate. The standard curve of peak area versus acid concentration was constructed over the range from 0.03 to 125 µg/mL and subjected to linear regression analysis (R^2^ = 0.9953). The calibration curve used for HPLC measurements is presented in Figure 1.

### 2.3. Assessment of Cytocompatibility of (Homo) and (Co)oligoesters Containing the p-Anisic Acid Moiety

#### 2.3.1. Statistical Analysis

The data were analyzed by use of a one-way analysis of variance (ANOVA) followed by a Tukey post hoc test. All of the obtained results were expressed as means ± SD. *p*-value of <0.05 was considered statistically significant. The analysis was performed with a use of Statistica 13.3 software (StatSoft, Kraków, Poland).

#### 2.3.2. SRB (Sulforhodamine B) Cell Proliferation Assay

The human keratinocyte HaCaT cell line (nontumorigenic, spontaneously immortalized cells) was purchased from Cell Lines Service (Eppelheim, Germany). Cells were cultured in DMEM (Dulbecco’s Modified Eagle’s Medium, Sigma-Aldrich, Chemie GmbH, Steinheim, Germany) containing 10% fetal bovine serum (PAN Biotech), 100 U/mL penicillin, 100 μg/mL streptomycin, and 20 mM HEPES (pH 7.3; Sigma-Aldrich, Chemie GmbH, Steinheim, Germany). Cell cultures were incubated at 37 °C in a humidified atmosphere with 5% CO_2_. The synthesized (co)oligoesters (samples 1–2) and unconjugated p-anisic acid were dissolved in dimethyl sulfoxide (DMSO) to obtain stock solutions. Working solutions were made by combining stock solutions with a complete culture medium, and sterile filtered. The final concentration range of the studied compounds was 1–100 µg/mL. DMSO concentration in every culture medium (including control) was adjusted to 0.2%. As a positive control, treatment with 5% DMSO in the culture medium was applied.

In Vitro Toxicology Assay Kit, Sulforhodamine B Based (Sigma-Aldrich, Chemie GmbH, Steinheim, Germany) was utilized to study cell proliferation. Keratinocytes were seeded into 96-well plates at an initial density of 3 × 10^3^ cells/well in 200 µL of medium. Cells were allowed to adhere and grow for 24 h. Then, the medium was replaced with working solutions, and keratinocytes were cultured for the next 72 h. After treatment, the culture medium was aspirated, and keratinocytes were fixed with 10% trichloroacetic acid, washed with deionized water, and stained with 0.4% SRB (sulforhodamine B) dissolved in 1% acetic acid. The unincorporated stain was washed out with 1% acetic acid and the incorporated sulforhodamine B was solubilized in 200 μL of 10 mM tris(hydroxymethyl)aminomethane solution. Absorbance was measured at 570 and 690 nm (reference wavelength) using the MRX Revelation plate reader (Dynex Technologies, Chantilly, VA, USA).

### 2.4. Hydrolytic Degradation Tests of Bioactive (p-AA-CH_2_-PL)_n_ Oligoester and [(p-AA-CH_2_-HP)_x_-co-(HB)_y_] (Co)oligoester With the p-AA Moiety

Hydrolytic degradation tests were performed, according to our previously elaborated protocol [21,23], the specimens were inserted into a thermo-regulated incubator (Memmert GmbH, Schwabach, Germany) set at 37 °C in deionized water (pH = 7) in 15 mL screw-capped vials with an air-tight Polytetrafluoroethylene (PTFE)/silicone septum in a period of 14 days. Approximately 20 mg of (p-AA-CH_2_-HP)_n_ or [(p-AA-CH_2_-HP)_x_-co-(HB)_y_] and 10 mL of aqua were mixed in glass vials. At different time intervals (24, 30, 48, 72 168, and 336 h), the aliquots were removed from the controlled environment and analyzed with the use of ESI-MS measurements accomplished in negative ion mode and by means of HPLC. The tests were carried out in triplicate.

## 3. Results and Discussion

We have recently developed an effective protocol for the preparation and characterization of (co)oligoesters containing bioactive moieties that were linked as pendant groups along oligomer backbones. The purpose of the current research was the preliminary assessment of the developed (co)oligoesters for their use as controlled DS for p-AA. The objects of this study were as follows: (p-AA-CH_2_-HP)_n_ (sample 1; see Section 2.1. Materials) oligoester and [(p-AA-CH_2_-HP)_x_-co-(HB)_y_] (co)oligoesters (sample 2, see Section 2.1. Materials) containing p-AA (as the bioactive end and side groups) connected to the polymer backbone through the susceptible to hydrolysis and ester bonds [25].

### 3.1. Characterization of (p-AA-CH_2_-HP)_n_ Oligoester Degradation Products

To evaluate the future use of synthesized (co)oligoesters as delivery and release systems for specific antioxidants, we examined hydrolytic degradation and the release profile of p-anisic acid. We used ESI-mass spectrometry for monitoring of hydrolytic degradation progress, identification of released p-anisic acid, as well as analysis of water fractions collected after defined hydrolysis time intervals that contained soluble in water degradation products formed during the hydrolysis process of the studied oligoesters.

**Figure 2:**
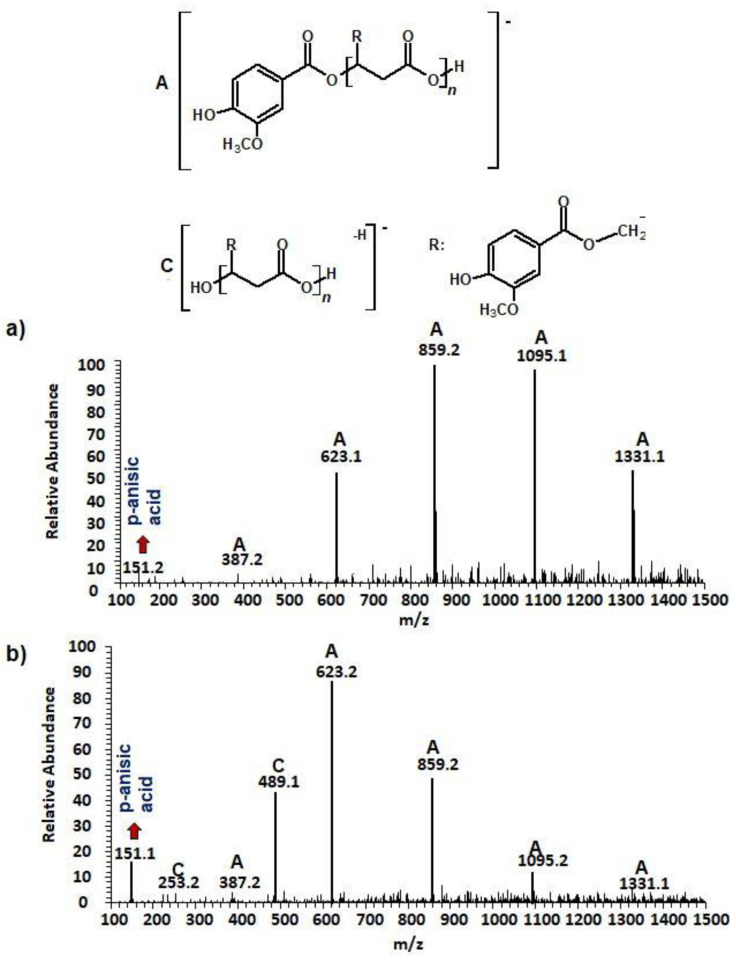
The negative electrospray (ESI) mass spectra of aqua solutions of the (p-AA-CH_2_-HP)_n_ (sample 1) (**a**) and medium collected after hydrolytic degradation (14 days) of the (p-AA-CH_2_-HP)_n_ at 37 °C together with chemical structures of the ions derived from series A and C (**b**).

Water solutions collected before (Figure 2a) and after 2 weeks (Figure 2b) of incubation of the (p-AA-CH_2_-HP)_n_ in water at a temperature of 37 °C were analyzed. ESI-mass spectra of the respective aliquots were acquired in the negative ion mode and are depicted in Figure 2.

One main series A of singly charged ions that occurred at 236 Da in a regular manner, which represent oligo(3-hydroxy-3-(4-methoxybenzoyloxymethyl)propionate) terminated by carboxylic and p-AA end groups, was observed in the mass spectrum depicted in Figure 2a. The shift of the distribution of ion patterns to the lower *m/z* values on the spectrum recorded after 2 weeks of the hydrolysis process was observed (Figure 2b). Furthermore, the intensity of the signals corresponding to oligoesters with lower molecular weights increased as a function of hydrolysis time (see Figure 2b). Thus, data obtained from ESI-MS analysis demonstrated that shorter (p-AA-CH_2_-HP)_n_ chains were formed. Additionally, the formation of oligo(3-hydroxy-3-(4-methoxy benzoyloxymethyl)-propionate) terminated by carboxylate and hydroxyl end groups (Series C) upholds the assumption that the hydrolysis process continued throughout the whole time-span regularly (Figure 2b). Moreover, the higher intensity signal at *m/z* 151 (corresponding to free p-AA) was observed, which confirms the hydrolysis process and release of the p-AA from the oligoester into the water fraction. The chemical structures of soluble in water degradation products (Series A and C) are depicted in Figure 2.

### 3.2. Characterization of [(p-AA-CH_2_-HP)_x_-co-(HB)_y_] (Co)oligoester Degradation Products

The chemical structure of [(p-AA-CH_2_-HP)_x_-co-(HB)_y_] (co)oligoesters, which were subjected to incubation in water after a specific period of time, was determined with the aid of ESI-MS analysis.

**Figure 3:**
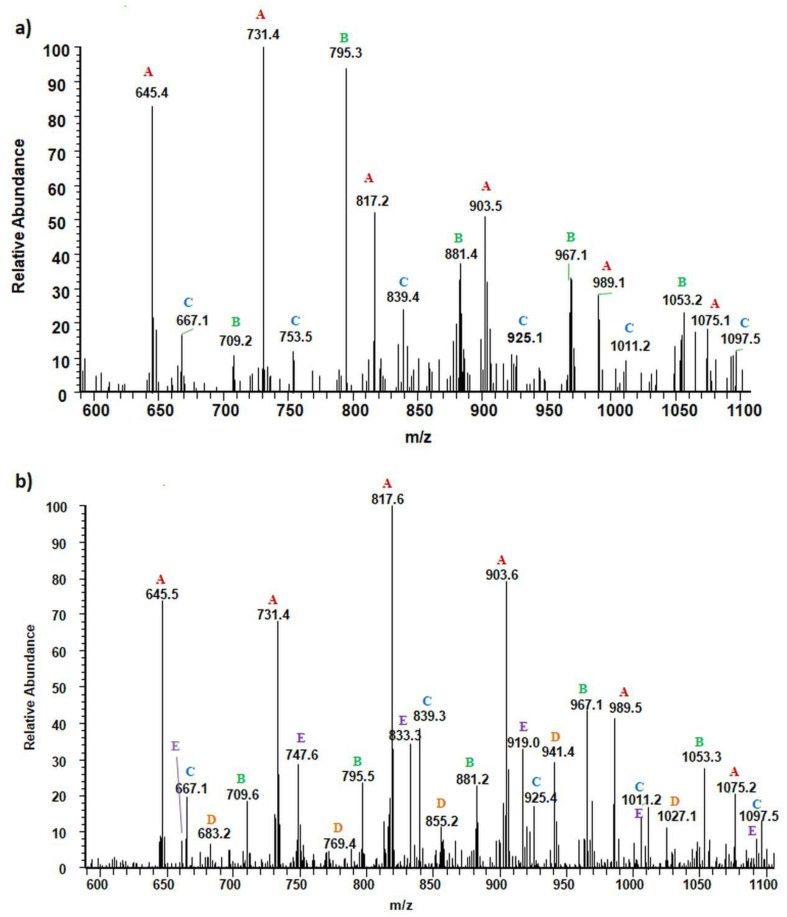
Spectral expansion in the range *m/z* 600–1100 of ESI-mass spectra (performed in negative ion-mode) of water solution of the [(p-AA-CH_2_-HP)_x_-co-(HB)_y_] (co)oligoesters collected before incubation (sample 2) (**a**) and aqua-soluble degradation products collected after hydrolytic degradation (14 days) of the [(p-AA-CH_2_-HP)_x_-co-(HB)_y_] (co)oligoesters at 37 °C (**b**).

Figure 3 shows the negative ESI mass spectrum in a spectral expansion in the range *m/z* 600–1100 of the water fractions before (Figure 3a) and after 2 weeks (Figure 3b) of incubation of the (co)oligoesters samples in water at 37 °C. The resulting ESI mass spectrum in the case of (co)oligoester consisted of a large number of ions and is much more complex owing to the various combinations of two (p-AA-CH_2_-HP) and (HB) comonomer units. To simplify the figures and Table 2, the abbreviation for the (p-AA-CH_2_-HP) comonomer unit was used, that is, (p-AA-CH_2_-HP)_x_ = A_x_. The peaks represented the individual (co)polyester chains at different polymerization degrees and their chemical composition has a tendency to group themselves in clusters. The major mass spacing that occurred between the signals belonging to the neighboring series was equal to 86 Da. These values correspond to the molecular weights of 3-hydroxybutyrate repeating units (86 Da).

Two main series of ions present in the ESI mass spectra, which appear at *m/z =* 151 + (*n* × 86) + 236 and *m/z =* 151 + (*n* × 86) + (2 × 236) (Series A and B, Figure 3a and Table 2), correspond to the (co)oligoester chains terminated by carboxylic and p-anisate end groups. The above mentioned (co)oligoester chains contain one (Series A) or two (Series B) (p-AA-CH_2_-HP) comonomer units arranged in a different combination with HB comonomer units along the oligomer backbone, (Series A) and (Series B), in Figure 3a and Table 2, respectively. In the mass spectra, we can observe a third and less abundant (with lower relative intensity) series of ions Series C (see Figure 3a and Table 2) at *m/z =* 151 + (*n* × 86). This series was ascribed to oligo(3-hydroxybutyrate) terminated by carboxyl and p-anisate moiety end groups. Table 2 contains the data with the ascribed structures of the ions presented in the ESI-MS spectra (Figure 3).

Additionally, in the mass spectra shown in Figure 3b, two series of anions, marked as Series D–E, were distinguished. These signals correspond to [(p-AA-CH_2_-HP)_x_-co-(HB)_y_] chains terminated by hydroxyl and carboxylate end groups, which were formed during the hydrolysis process of the (co)oligoester studied. However, the formation of oligo-3-hydroxybutyrate terminated by carboxylate and hydroxyl end groups was not detected in the spectrum. Most probably, the lack of the detection of these signals was due to too low intensity of peaks, below the detection limit. The chemical structures of the identified oligomers (Series A–E) are depicted in Table 2.

A deeper knowledge of the structure of individual (co)oligoester chains was gained with the use of tandem ESI-MS/MS. This technique was also previously applied by us for structural studies of individual molecular ions selected from different types of copolymers and allowing differentiation of the individual molecular chains of the random and diblock copolyesters [27,28].

**Figure 4:**
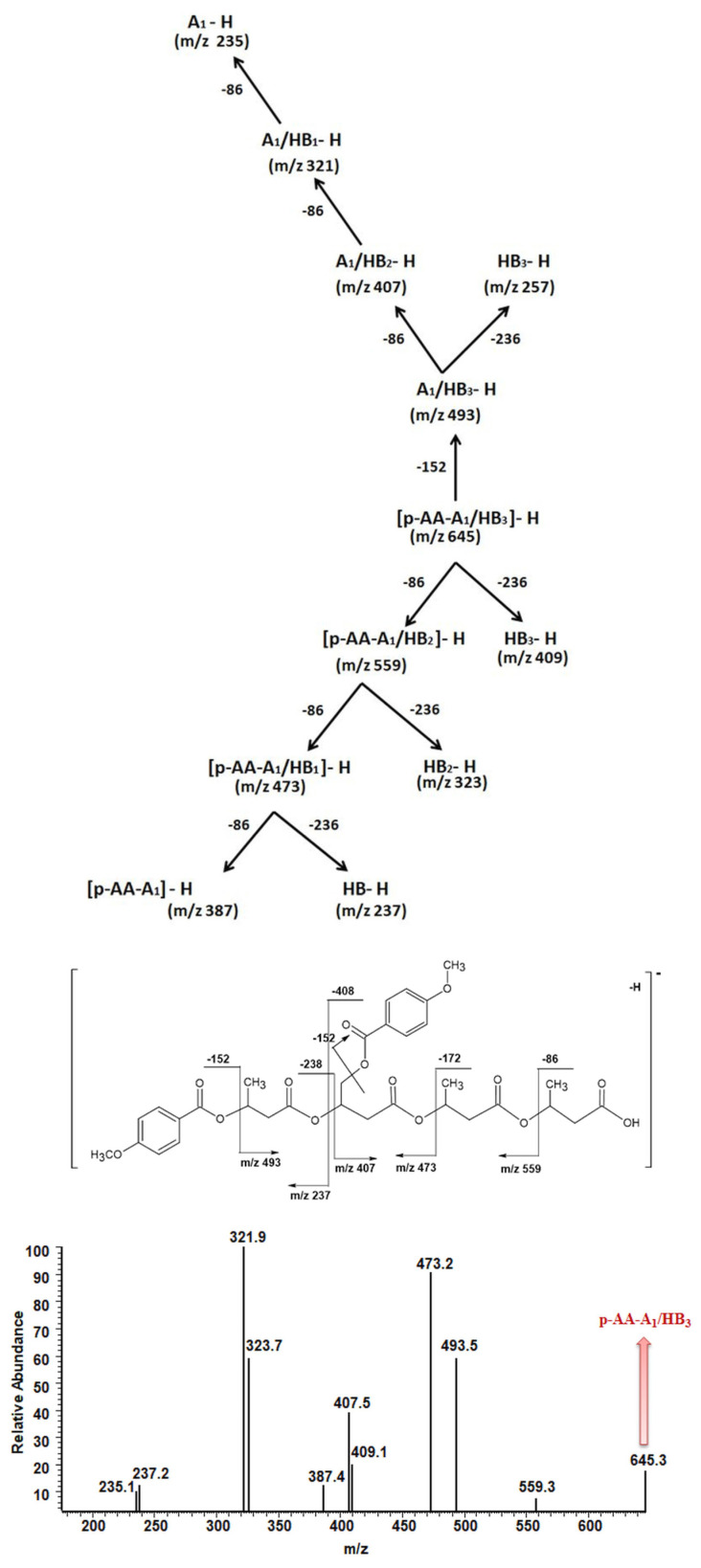
ESI-MS^2^ spectrum (performed in negative-ion mode) of [p-AA-A_1_/HB_3_] (co)oligoester ion at *m/z* 645 with carboxylic and p-anisate end groups and possessing one p-AA-CH_2_-HP comonomer unit together with the theoretical fragmentation pathway.

We applied tandem mass spectrometry (ESI-MS/MS) in order to confirm the structural assignment of the ions present in the obtained ESI-MS spectra. In particular, the acquired data were crucial to verify the distribution of p-AA-CH_2_-HP and HB comonomers along the [(p-AA-CH_2_-HP)_x_-co-(HB)_y_] (co)oligoester chains. Herein, we show as an example ESI-MS/MS spectrum (in negative ion mode) of the precursor ion at *m/z* 645, representing oligomers terminated by carboxyl and p-anisate moiety end groups. The verified ion of interest contained three 3-hydroxybutyrate repeating units and one p-AA-CH_2_-HP unit distributed randomly in the (co)oligoester chain. The fragment ion at *m/z* 493 corresponds to the oligomer formed by the loss of p-AA (152 Da), which can be derived from the terminal group and/or from the pendant group of the p-AA-CH_2_-HP comonomer unit. The product ion at *m/z* 559 represents the oligomer formed by the loss of the 3-hydroxybutyrate unit (crotonic acid; 86 Da) in the oligoester chains. The product ion at *m/z* 409 corresponds to the oligomer formed by the loss of the last, in the oligoester chain, p-AA-CH_2_-HP comonomer unit in the form of 4-methoxybenzoyloxycrotonic acid (236 Da, see Scheme in Figure 4). The ESI-MS/MS spectrum and proposed fragmentation pathway clearly confirm that the p-AA-CH_2_-HP comonomer units are arranged in a random manner in the oligomer chain.

Fragmentation of the precursor ion at *m/z* 645 (Series A, Figure 3 and Table 2) proceeds as a result of random cleavage of ester bonds along the oligomer chain and ester bonds between the chain and of p-anisate pendant group. In the case of solutions containing both acids and oligomers, the intensity of signals in the ESI spectra corresponding to acid molecules is lower than in the case of oligomers [29]. For a quantitative estimation of (co)oligoesters and acid content, based on ESI mass spectra obtained for mixtures, a separate calibration for each type of ingredient is required. Therefore, the release profile of p-anisic acid was determined separately by HPLC (see Section 3.3, *Comparative studies of the release of p-anisic acid from* (p-AA-CH_2_-HP)_n_ *oligoester and [(p-AA-CH_2_-HP)_x_*-co-*(HB)_y_] (co)oligoesters*)).

### 3.3. Comparative Studies of the Release of p-Anisic Acid from (p-AA-CH_2_-HP)_n_ Oligoester and [(p-AA-CH_2_-HP)_x_-co-(HB)_y_] (Co)oligoesters

**Figure 5:**
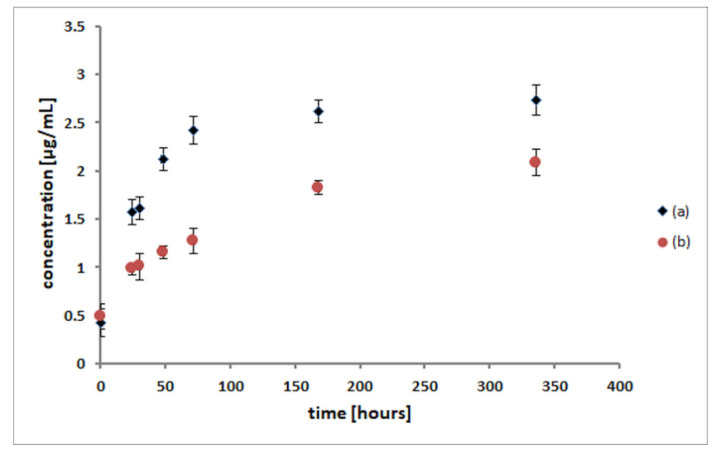
Comparative release profiles of p-anisic acid from (p-AA-CH_2_-HP)_n_ homo(oligoester) (**a**) and [(p-AA-CH2-HP)_x_-co-(HB)_y_] (co)oligoesters (**b**).

The release studies of p-anisic acid from both (p-AA-CH_2_-HP)_n_ oligoester and [(p-AA-CH_2_-HP)_x_-co-(HB)_y_] (co)oligoesters were performed in aqua solutions at 37 °C for the period of 14 days. To determine the amount and profile of the p-anisic acid release from [(p-AA-CH_2_-HP)_x_-co-(HB)_y_] copolymers, the mediums collected after a specific period of degradation of (co)oligoesters were analysed by high-performance liquid chromatography (HPLC) equipped with a DAD detector. The results of HPLC analysis, including the presence of p-anisic acid and calculated based on the calibration curve of the amount of acid released from the studied (co)oligoesters over time, are presented in Figure 5.

The amount of p-AA released from (p-AA-CH_2_-HP)_n_ oligoester and [(p-AA-CH_2_-HP)_x_-co-(HB)_y_] (co)oligoesters was determined for 14 days. Initially, a slightly higher release rate was observed, but after 3 days, p-AA showed a relatively slower release rate. From the p-AA release profile, it is clear that almost 50% of the bioactive compound studied was released from the carrier in the first 3 days in the case of [(p-AA-CH_2_-HP)_x_-co-(HB)_y_] and, in the case of (p-AA-CH_2_-HP)_n_, it was more than 75%. Additionally, we consider such concentrations as achievable in the epidermis, taking into account possible cosmetic formulations, as well as release and absorption rates described in our previous work in this field [23]. We should highlight that the release of a bioactive agent proceeded regularly. However, the release rate of p-AA was much faster for (p-AA-CH_2_-HP)_n_, which is an expected result as this carrier possessed a higher loading of p-AA per polymer macromolecule compared with (co)oligoester carrier (Figure 5). As we described previously, bioactive oligoesters that contain from 1 to 8 of p-AA molecules and (co)oligoesters that contain from 2 to 3 molecules of the p-AA moiety (as the bioactive end and side groups) were synthesized [25].

### 3.4. Cytocompatibility of (Homo)- and (Co)oligoesters Containing p-AA Moiety

#### SRB Assay for Cell Proliferation

**Figure 6:**
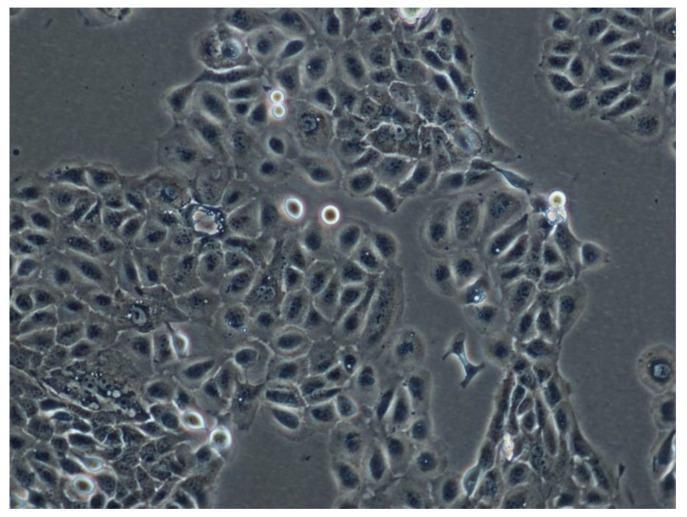
Phase-contrast micrograph of HaCaT keratinocytes in culture flask (original magnification: ×100).

To study the cytocompatibility of the synthesized (homo)- and (co)oligoesters with biosystems, their effects on cell growth were measured using sulforhodamine B staining. The HaCaT cell line was derived from normal human epidermis and the cells underwent spontaneous immortalization during successive passages [30]. These cells are not tumorigenic and they retained some properties of normal keratinocytes such as contact inhibition of proliferation or the ability to differentiate [31]. Therefore, the HaCaT cell line has become a popular model for testing dermal drugs and cosmetics. In the culture flask, this cell line grows as a monolayer of densely packed cells (Figure 6). The substances tested in our study were applied over a relatively wide concentration range (1–100 µg/mL). The concentration range of p-anisic acid (and its conjugates) was set up based on data referring to the biological activities of that compound. It has been shown to influence the enzymatic activity of proteasome and lysosomal cathepsins at such low concentrations as 5 µM (0.76 µg/mL) [32]. Inhibition of tyrosinase activity was detectable from 0.1 mM (15 µg/mL), with an IC50 value 0.6 mM (91 µg/mL) [33]. Antiproliferative activity of p-anisic acid against HepG2 cancer cell line was observed for the concentration range of 1–100 µM, with IC50 coming to 25 µM (3.8 µg/mL) [34]. On the other hand, we consider such concentrations as achievable in the epidermis, taking into account possible cosmetic formulations, as well as release and absorption rates [23].

**Figure 7:**
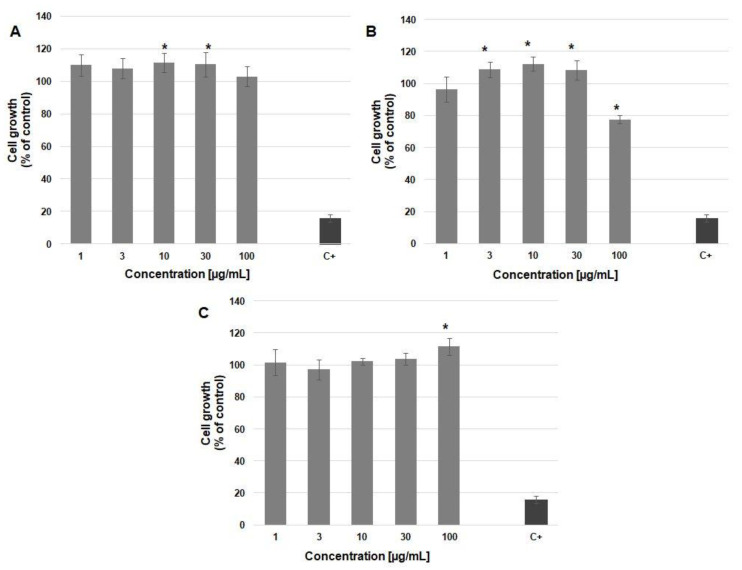
Growth of HaCaT cells in the presence of (**A**) (p-AA-CH_2_-HP)_n_; (**B**) [(p-AA-CH_2_-HP)_x_-co-(HB)_y_]; and (**C**) p-anisic acid. Each bar represents the mean ± SD; * *p* < 0.05 versus the control group (analysis of variance, ANOVA); C+—positive control.

As shown in Figure 7A, (p-AA-CH_2_-HP)_n_ did not exert any detrimental effect on keratinocyte proliferation. In fact, in treated wells, average cell numbers were greater than in the control. The statistical analysis revealed significant differences in cultures treated with (p-AA-CH_2_-HP)_n_ at concentrations of 10 and 30 µg/mL. As shown in Figure 7C, free p-anisic acid exerted a significant cell growth promotion effect at the concentration of 100 µg/mL. Mechanisms responsible for that effect are unknown as only scant information exists on the p-methoxybenzoic acid impact on human cell functions. It has been previously suggested that the conjugates of drugs and oligo-(R,S)-(3-hydoxybutyrate) (OHB) are more efficiently taken up by cells, compared with non-conjugated molecules [35]. More efficient accumulation inside cells would explain the ability of our (homo)oligoester to stimulate keratinocyte proliferation at lower concentrations, compared with the free acid.

A pattern of keratinocyte response to [(p-AA-CH_2_-HP)_x_-co-(HB)_y_] was slightly different (Figure 7B). That is, (co)oligoester increased cell proliferation at concentrations of 3, 10, and 30 µg/mL, but inhibited cellular growth at the highest dose (100 µg/mL). In our previous research [23], we demonstrated some growth inhibitory properties of oligo(3-hydroxybutyrate) carriers in HaCaT cells. Zawidlak-Wegrzynska et al. [35] demonstrated that conjugation of ibuprofen with OHB increased its antiproliferative effects in colon cancer HT-29 and HCT 116 cell lines. OHB oligomer alone also exhibited some growth inhibitory action, though at relatively large concentrations. Oligo(3-hydroxybutyrate) loaded into liposomes reduced the growth of L929 cells and induced cell death and cell cycle arrest at the G_1_/G_0_ phase [36]. On the basis of these observations, we suggest that the presence of HB units in the [(p-AA-CH_2_-HP)_x_-co-(HB)_y_] chain and somewhat greater molecular mass could possibly favour the revealing of its antiproliferative activity in the present study. The mechanism underlying the antiproliferative effect of OHB is currently unknown. However, it is worth pointing out that 3-hydroxybutyrate dose-dependently decreased proliferation of the human kidney HK-2 cell line [37]. It was accompanied by cell cycle arrest at the G_0_/G_1_ phase and increased p21^WAF1^ as well as p27^kip1^ protein expression. These changes were mediated by oxidative stress, Smad3, and TGF-β. It has been shown that mammalian cells or body fluids (as serum) are able to hydrolyse dimers and trimers of 3-hydroxybutyrate owing to the action of enzymes, for example, carboxylesterase [38]. Therefore, 3-hydroxybutyrate could mediate the growth inhibitory effects of [(p-AA-CH_2_-HP)_x_-co-(HB)_y_]. After all, mild inhibition of HaCaT cell growth was seen solely at a very high concentration of the studied (co)oligoester, which allows considering it biocompatible. In practice, it is advisable to carefully adjust its concentration in cosmetic preparation to obtain an optimal effect on epidermal cells.

## 4. Conclusions

With regard to likely applications of the developed (p-AA-CH_2_-HP)_n_ and [(p-AA-CH_2_-HP)_x_-co-(HB)_y_] (co)oligoesters in the area of biomaterials, especially in cosmetology, comprehensive in vitro cytotoxicity tests as well as hydrolytic degradation under laboratory conditions were performed.

The application of ESI-mass spectrometry to characterize aqua-soluble hydrolytic degradation products of (p-AA-CH_2_-HP)_n_ oligoesters and [(p-AA-CH2-HP)_x_-co-(HB)_y_] (co)oligoesters allowed us to determine their molecular level chemical structure and helped us to confirm the release of bioactive p-AA from the developed systems.

Biological comparative in vitro studies showed that the synthesized (co)oligoesters were non-toxic and were well tolerated by the HaCaT cells. Both (homo)- and (co)oligoesters exerted a beneficial effect on keratinocyte growth, especially at moderate concentrations. The highest concentration of the (co)oligoester caused only a mild inhibition of cell proliferation. The presented study shows the potential of the developed (homo)- and (co)oligoesters as novel controlled release and delivery systems for future applications in the cosmetics industry.

## Figures and Tables

**Table 1:** Solvent gradient used for high-performance liquid chromatography (HPLC) analysis.

Time (min)	Mobile Phase (%)	
	A	B
0	75	25
5	75	25
20	60	40
25	55	45
45	45	55
50	75	25

A is 0.1% formic acid in water and B is acetonitrile.

**Table 2:** Structural assignments of ions present in the enlarged region (*m/z* 600–1100) of the electrospray mass spectrometry (ESI-MS) of the degradation products of [(p-AA-CH_2_-HP)_x_-co-(HB)_y_]. Spectra depicted in Figure 3 and Figure 4.

Series	Chemical Structure of the Ions	Composition of the Comonomers	*m/z*
**A**	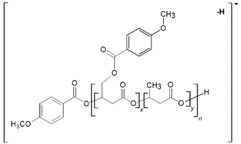	(p-AA-CH_2_-HP)_x_ = A_x_	
		p-AA-A_1_/HB_3_	645
		p-AA-A_1_/HB_4_	731
		p-AA-A_1_/HB_5_	817
		p-AA-A_1_/HB_6_	903
		p-AA-A_1_/HB_7_	989
		p-AA-A_1_/HB_8_	1075
**B**	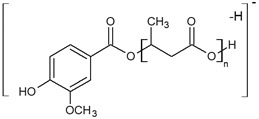	p-AA-A_2_/HB_1_	709
		p-AA-A_2_/HB_2_	795
		p-AA-A_2_/HB_3_	881
		p-AA-A_2_/HB_4_	967
		p-AA-A_2_/HB_5_	1053
**C**	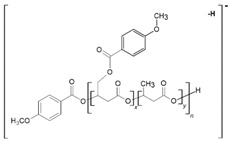	p-AA-HB_6_	667
		p-AA-HB_7_	753
		p-AA-HB_8_	839
		p-AA-HB_9_	925
		p-AA-HB_10_	1011
		p-AA-HB_11_	1097
**D**	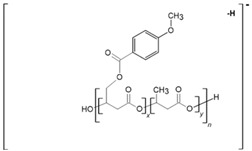	A_1_/HB_5_OH	683
		A_1_/HB_6_OH	769
		A_1_/HB_7_OH	855
		A_1_/HB_8_OH	941
		A_1_/HB_9_OH	1027
**E**	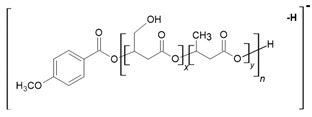	A_2_/HB_2_OH	661
		A_2_/HB_3_OH	747
		A_2_/HB_4_OH	833
		A_2_/HB_5_OH	919
		A_2_/HB_6_OH	1005
		A_2_/HB_7_OH	1091
